# The Benjamin H. Kean Travel Fellowship in Tropical Medicine: Assessment of Impact at 15 Years

**DOI:** 10.4269/ajtmh.17-0120

**Published:** 2017-06-26

**Authors:** Aubri S. Carman, Chandy C. John

**Affiliations:** 1 Ryan White Center for Pediatric Infectious Disease and Global Health, Indiana University School of Medicine, Indianapolis, Indiana;; 2 The University of Arizona College of Medicine, Tucson, Arizona

## Abstract

The Benjamin H. Kean Fellowship in Tropical Medicine is an American Society of Tropical Medicine and Hygiene initiative that provides medical students with funding for international clinical or research experiences lasting at least 1 month. Of the 175 Kean fellows from 1998 to 2013, 140 had current available e-mails, and 70 of the 140 (50%) responded to a survey about their fellowship experience. Alumni indicated that the Kean Fellowship had a high impact on their career plans with regard to preparation for (*N* = 65, 94.2%) and inspiration to pursue (*N* = 59, 88.1%) a career in tropical medicine and global health. Continued involvement in tropical medicine and global health was common: 52 alumni (74.3%) were currently working in tropical medicine or global health, 49 (71.0%) had done so in the interim between the Kean fellowship and their current position; and 17 of 19 Kean fellows (89.4%) who had completed all medical training and were now in professional practice continued to work in tropical medicine and global health. Alumni had been highly productive academically, publishing a total of 831 PubMed-indexed manuscripts, almost all on tropical medicine or global health topics, in the period between their fellowship year and 2013. Alumni reported strengths of the fellowship including funding, networking, and flexibility, and suggested that more networking and career mentoring would enhance the program. The Benjamin H. Kean fellowship program has been highly successful at inspiring and fostering ongoing work by trainees in tropical medicine and global health.

## INTRODUCTION

Medical students in the United States continue to indicate increasing interest in the study of tropical medicine and global health (TMGH) and opportunities for international experiences in the field during their medical education.^1–6^ Previous studies have described the rapidly increasing number of medical students who complete international experiences—in 1984, just 6% of medical students had such an experience compared with 29% of graduating medical students surveyed in 2014.^1,4^ Students who completed international elective experiences were more likely to pursue careers in primary care or public service, have increased confidence in physical examination and history taking skills, and both suspect and recognize diseases endemic to the developing world in either immigrants or traveling patients at their home institutions.^5,7–9^ International elective experiences have also been linked to enhancing students’ desire to work with underserved populations, a desire that normally suffers a well-documented decline among medical trainees throughout the continuum of pre- and postgraduate education.^7^ The benefits of international experience are vast and timely given the current pace at which globalization is occurring.

Evaluations of the Fogarty Scholars and Fellows program—a program in which participants spend 10 months or more abroad in a primarily research capacity—have found that trainees’ experiences abroad shape their future career plans and many are likely to pursue careers in the TMGH fields.^2,10,11^ Investment in international experiences for trainees yields returns in terms of shaping the future TMGH workforce as evidenced by increasing importance of global health training availability in the selection of a residency program.^12,13^

Despite the increasing demand for international experiences for trainees, medical education and residency training programs have been slow to respond.^5,9^ In a comprehensive analysis of global health education in medical schools, Drain and others found that “currently, the limited number of opportunities and difficulty in arranging an international rotation discourage medical students from expanding their clinical experience.”^5^ The most commonly cited barrier to student participation in such international experiences is funding; 90% of students surveyed at Johns Hopkins School of Medicine indicated that financial assistance was important in influencing their decision to go abroad.^1,5^ Many students must turn to outside funding sources to have the opportunity to pursue these beneficial international experiences.

The American Society of Tropical Medicine and Hygiene (ASTMH) started the Benjamin H. Kean Travel Fellowship in Tropical Medicine in 1998, to provide support for North American medical students to conduct research or gain clinical care experience in tropical medicine. The Kean fellowship was started to honor Benjamin Kean, a long-time member of ASTMH and a legendary clinician and educator in parasitology at the Weill Cornell Medical College. Among his many accomplishments in clinical research, Kean was perhaps best known for discovering that enterotoxigenic *Escherichia coli* was the cause of traveler’s diarrhea. Stephen Hoffman, one of Kean’s former students, headed the effort to raise funds for the award, and members of American Committee of Clinical Tropical Medicine and Traveler’s Health ( also known as the Clinical Group) were key contributors to the fund. The fellowship was started in 1998, under the leadership of Christopher Plowe, also a former student of Kean’s, and four awards were given that year. Over the next several years, between four and 12 awards were given. In 2010, with greater yield from fund resources, the number of fellows increased to 20 per year, a number of fellowships that has remained relatively stable through 2016.

In line with the goals of the Society, the fellowship seeks to encourage young trainees to continue work in tropical medicine and hygiene as well as strengthen ties between past leaders in the field and the next generation of leaders. Fellows are afforded the cost of a round-trip flight to their destination and $1,000 toward living expenses. As part of a rigorous selection process, medical students are required to submit transcripts, CV’s, and recommendation letters in addition to responses to brief essay questions. In line with alleviating the ethical concerns of short-term experiences, students must also submit information about their proposed elective site, and must commit to at least 1 month at the site. Those applying to use the fellowship for research funding must submit an abbreviated proposal of their project and quality evidence of prior contact and extensive discussion with their overseas mentor.^14^ In the past 5 years, 35–45 students have applied every year, and 18–22 students receive a fellowship. Kean Fellows come from medical schools all over the country, and have conducted research or gained clinical experience in numerous different low and middle income country (LMIC) medical institutions.

In 2013, the senior author of this study (CCJ), as chair of the Kean Fellowship committee, together with the Executive Director of ASTMH, Karen Goraleski, developed a survey of fellows who had completed their fellowships during the first 15 years of the fellowship, from 1998 to 2013. The objective of the survey was to assess the effectiveness of the fellowship in providing opportunities for medical students to go abroad, and both if and how past fellows remain engaged in TMGH. The Society also aimed to solicit feedback about the fellowship’s strengths and perceived areas in need of improvement. The survey was for quality improvement and program evaluation and was not considered research or in need of research review by the Indiana University Institutional Review Board.

## METHODS

ASTMH and Kean Fellowship staff retrospectively collected data using an electronic 17-question survey. A link to complete the survey was e-mailed to alumni who had received the Kean Fellowship from 1998 to 2013. A single follow-up reminder was sent to fellows who did not complete the survey. The survey asked alumni to report details about their current career positions as well as current and past involvement in TMGH. Multiple responses/categories were allowed to demonstrate the breadth of TMGH work. Respondents were also asked to indicate the impact that the Kean Fellowship had on the role of TMGH in their career and pursuit of future TMGH opportunities. Additionally, respondents were asked to comment regarding the strengths and possible areas of improvement for the fellowship through open-field response questions. The survey questions are summarized in the tables and figures in the Results section, and the full list of questions is listed in Supplemental Information: List of Questions for Kean Fellow Survey. Survey responses were anonymized before review.

Descriptive statistics were used to quantify responses. The text of responses to open-field questions was coded line by line using a codebook generated by ASC. Content thematic approach was then used to extract important themes and trends among the responses, and selected quotes are provided to illustrate the findings of this analysis. StataSE v. 12 (College Station, TX) was used to analyze quantitative data.

## RESULTS

### Respondents.

Of 175 eligible alumni, 140 had functional e-mail addresses. We received 70 responses, for a response rate of 50%. As demonstrated in Table 1, most respondents were currently trainees, as medical students, residents, and fellows composed approximately 67% of respondents (27.1%, 27.1%, and 12.9%, respectively). Accordingly, most respondents (88.6%) were currently working or studying in an academic setting. Nineteen respondents (27.1%) had completed training and were practicing clinicians. Of the respondents that had completed training, 13 (68.4%) were working in an academic setting, four (21.1%) in a community practice, and two (10.5%) in government agencies. There were no specific questions about the field in which respondents were training or practicing, but those who provided such information in the context of other responses indicated specialties in emergency medicine, internal medicine, pediatrics, cardiovascular medicine, infectious disease, hematology, dermatology, pathology, family medicine, medical microbiology, and pediatric infectious disease. Table 2 provides a list of locations where Kean Fellows from 1998 to 2013 worked during their fellowship, organized by region. (Information on country of fellowship was missing for eight fellows, so the total number of fellows in Table 2 is 167).

**Table 1:** Respondent characteristics

Characteristic	Respondents *N* = 70 *n* (%)
Current position	
Medical Student	19 (27.1)
Resident	19 (27.1)
Fellow	9 (12.9)
Clinician-professor/instructor	11 (15.7)
Clinician-general	8 (11.4)
Other research position (graduate student, research assistant, postdoctoral fellow)	4 (5.7)
Type of practice	
Academic medical center	62 (88.6)
Government agency	4 (5.7)
Community	4 (5.7)

**Table 2:** Destinations for Work during Kean Fellowship 1998–2013

Africa		Central and South America		Asia	
Country	Fellows *N* = 87 (52.1%)	Country	Fellows *N* = 47 (28.1%)	Country	Fellows *N* = 33 (19.8%)
Angola	1	Argentina	3	Bangladesh	4
Benin	1	Bolivia	3	Cambodia	1
Bostwana	2	Brazil	10	India	6
Burkina Faso	1	Colombia	1	Indonesia	1
Cameroon	1	Ecuador	2	Laos	1
DR Congo	1	El Salvador	1	Myanmar	2
Egypt	2	Guatemala	4	Nepal	4
Ethiopia	4	Haiti	3	Philippines	1
Gabon	1	Honduras	2	Sri Lanka	2
Gambia	2	Nicaragua	1	Thailand	11
Ghana	7	Panama	2		
Kenya	11	Peru	15		
Madagascar	1				
Malawi	8				
Mali	3				
Niger	1				
Rwanda	2				
Senegal	1				
Sierra Leone	1				
South Africa	5				
Tanzania	6				
Uganda	20				
Zambia	3				
Zimbabwe	2				

### Career path influence.

**Figure 1.**
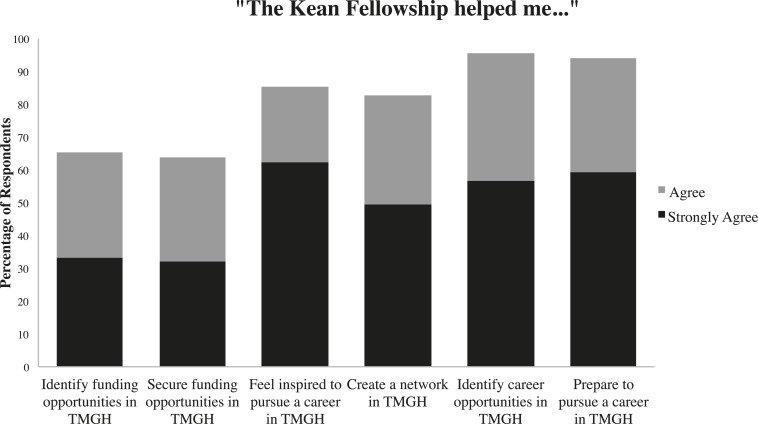
Alumni were asked the extent to which they agreed with a set of statements regarding their experience as Kean fellows. The statements, all beginning with “The Kean Fellowship helped me…,” are presented on the horizontal axis with corresponding graphic representation of percentages of positive alumni responses.

Based on alumni responses, the Kean fellowship appears to be highly influential in helping trainees to identify and pursue a career in TMGH. As shown in Figure 1, almost all respondents (94.2%) agreed that the Kean fellowship helped prepare them to pursue a career in TMGH. More than 80% of fellows agreed that their time as a Kean fellow not only inspired them to pursue TMGH in their careers, but also helped them to network and identify career opportunities in the field. Smaller percentages of the fellows agreed that the fellowship helped them to identify funding opportunities in TMGH (65.2%), and that the fellowship helped to secure such funding opportunities (63.8%). A PubMed search of the 175 Kean alumni from 1998 to 2013, using specific identifiers to insure that individuals with similar names were not counted in the search, demonstrated the high academic productivity of Kean fellows. From the time of their fellowship year to 2013, Kean fellowship alumni published 831 papers, almost all of which related to TMGH topics. The average number of published papers per Kean fellow was 4.75, and although specific fellows did play an outsize role in publication productivity (nine fellows published 30 or more papers, and one published 81 papers), 65% of fellows had published at least one manuscript in the period since their fellowship award.

### Current and interim TMGH engagement.

The retention rate of fellows in TMGH work was high. Almost three-quarters of respondents indicated they were engaged in TMGH work at the time of the survey (*N* = 52, 74.3%), and a similar number of respondents (*N* = 49, 71.0%) indicated that they had been active in TMGH work in the interim period between the completion of their Kean fellowship and their current position. Of the 19 Kean Fellows who had completed training (i.e., completed residency or fellowship), 17 (89%) were still active in TMGH work, an encouraging sign of the long-term commitment of Kean fellows to TMGH.

**Figure 2.**
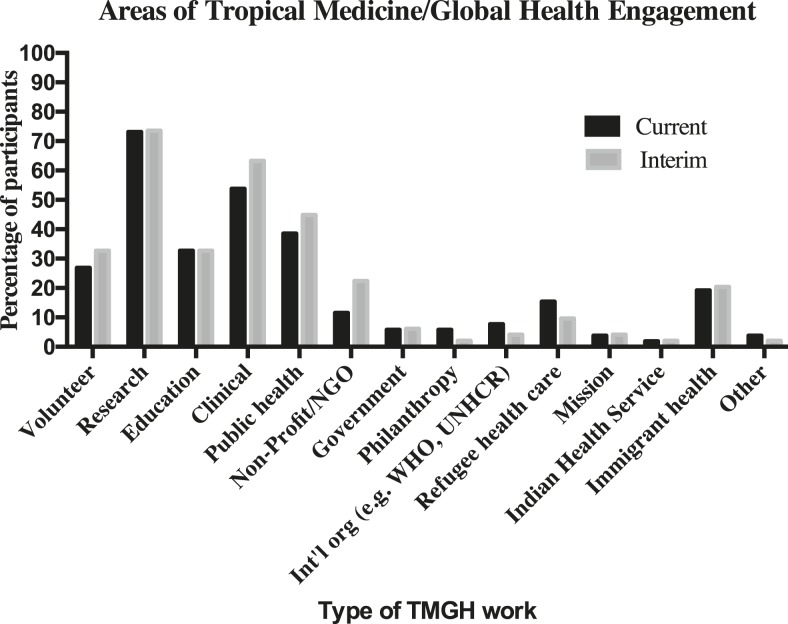
Alumni responses regarding current and past involvement in tropical medicine and global health.

As shown in Figure 2, both current and interim TMGH work was most commonly research (73.1% and 75%, respectively) or clinical (53.8% and 64.6%, respectively) in nature. Almost one-quarter (24.6%) of respondents were working in LMICs, with most of these projects in Africa (*N* = 13, 76.5%), and a few in Asia (*N* = 2, 11.8%) and South/Latin America (*N* = 1, 5.9%). Approximately two-thirds of respondents (*N* = 40, 61.5%) had done international work in the interim period between completing their fellowship and being in their current position. The majority of respondents indicated they had 1–6 months of international work experience during this time (63.4%), with 12.2% of respondents having experience that lasted 2 or more years. Again, the most common location for interim work was on the African continent (*N* = 26, 65.0%), followed by South/Latin America (*N* = 17, 42.5%), and Asia (*N* = 8, 20.0%). Ten respondents (14.3%) indicated past involvement in more than one region.

Notably, many alumni were also involved in domestic TMGH activities such as refugee health, immigrant health, and the Indian Health Service. Nearly 37% of alumni were currently engaged in these types of TMGH, whereas 33.3% reported that they had been engaged in such activities in the interim period. These results show that Kean alumni demonstrate meaningful engagement in local health systems as well as international projects.

### Fellowship reflections.

Over three-quarters (77.1%) of survey respondents offered feedback regarding the strengths of the Kean Fellowship, whereas a smaller percentage offered feedback about suggestions for program improvement. Coded responses to these open-field questions are provided in Table 3.

**Table 3:** Strengths and Suggested Areas of Improvement for the Kean Fellowship

Strengths of the Kean Fellowship	
Q: In your opinion, what are the strengths of the ASTMH Benjamin Kean Fellowship? (Open response, 54 responses)	*n* (%)
Funding	20 (37.0)
The opportunity to do tropical medicine work	15 (27.8)
Networking with others in the field	13 (24.1)
Flexibility in time and site	12 (22.2)
Target audience of medical students	7 (13.0)
Requirements are feasible	5 (9.2)
Administration of the fellowship by ASTMH	5 (9.2)
Influences fellow’s career choice	3 (5.6)
Ease of the application process	2 (3.7)
Other	1 (1.9)
Aspects of the Kean Fellowship that should not be changed	
Q: In your opinion, what aspects or components of the ASTMH Benjamin Kean Fellowship should definitely NOT be changed? (Open response, 20 responses)	*n* (%)
Funding	8 (55.0)
Target audience of medical students	5 (25.0)
Flexibility in time and site	4 (20.0)
Networking opportunities	3 (15.0)
Overall goal	2 (10.0)
Ease of the application process	2 (10.0)
Requirements are feasible	1 (5.0)
Administration of the fellowship by ASTMH	1 (5.0)
Suggestions to improve the Kean Fellowship	
Q: What suggestions do you have to improve the program for future participants? (Open response, 30 responses)	*n* (%)
More inter-fellow networking	12 (40.0)
Expansion to include more fellowships	4 (13.3)
More follow-up and career mentorship	10 (33.3)
More supervision	3 (10.0)
Longer time requirement	3 (10.0)
Other	2 (6.7)
Award date	1 (3.3)
ASTMH conference	1 (3.3)

ASTMH = American Society of Tropical Medicine and Hygiene.

Most commonly, alumni identified the funding provided as both a strength of the fellowship (37.0%) and an aspect of the fellowship that should not change (55.0%). Many respondents noted, unsurprisingly, that often the financial burden of TMGH was preventative, and the fellowship helped to alleviate that burden. As one respondent said, “[The Kean Fellowship provided me with] a good level of funding without restrictions that allowed me to fully explore my interest in global public health.” Others specifically cited the networking benefits of the fellowship (24.1%), the flexibility with regard to both time and site (22.2%), and the fact that the fellowship targets medical students (13.0%) as strengths of the fellowship. Presumably, many of these factors are also represented by fellows who responded that the general “opportunity to do TMGH work” was a large strength of the fellowship (27.8%). “The chance to do a meaningful project abroad is a huge strength,” said one fellow. Another noted that the fellowship “Gave me the opportunity to strengthen my insight of the global issues at hand.” A fair number of respondents highlighted the target audience of the fellowship—medical students—as something that should not change. “Keep [the fellowship] open to those with little experience but keen interest,” said one respondent.

Suggestions for improvement largely focused on expansion of various aspects of the Kean Fellowship program. Many respondents cited that there should be more networking among fellows (40.0%) as well as more follow-up and career mentorship (33.3%). Lack of funding for travel to the ASTMH annual meeting was a barrier to attendance among alumni. Fellows were interested in more extensive networking among themselves as well as with others in the TMGH field. As one fellow said, “My only interaction with the fellowship was obtaining funding. It was really nice to have no strings but maybe that was a lost opportunity. There was no other requirement to present my work, network, go to meetings, etc. Maybe require a presentation or conference attendance by the fellows.”

## DISCUSSION

The Kean fellowship survey results show that the fellowship has succeeded in its goals of inspiring interest and fostering ongoing work by North American medical students in TMGH. The Kean fellowship provided the needed resources for these medical students to pursue a tropical medicine rotation, and this experience and their experience with other Kean fellows and ASTMH members appears to have encouraged continued work in TMGH. Fellows remained involved in TMGH work in a variety of capacities, showing that the ASTMH investment in early career trainees through this fellowship is paying dividends not only internationally, but also in local health systems through engagement in refugee, immigrant, and Indian health. Perhaps most encouragingly, Kean fellows have been highly academically productive, with 831 postfellowship publications from 1998 to 2013, and retained a strong commitment to TMGH, with ∼90% of those now in professional practice continuing to be involved in TMGH work.

Most felt that the Kean Fellowship provided an early opportunity for TMGH study that otherwise may not have been possible. As described previously, many medical students cite funding as the largest barrier to completing international electives, and our results demonstrate that Kean Fellowship helps to alleviate this barrier in a meaningful way. The Kean Fellowship is one of few funding sources available for medical students to conduct short-term TMGH work. In response to increasing demands in global health training opportunities, Kerry and others have suggested that global health funding should be channeled to sustained, rational, and effective training programs to have the most impact—criterion that the Kean Fellowship appears to uphold based on survey responses.^13^ Further investment in this and similar programs would continue to enhance the field of TMGH professionals by nurturing interest in TMGH early in training and providing more outlets for networking and mentoring within the field.

Alumni indicated that the Kean Fellowship had a variety of strengths not only centered on funding and opportunity, but also encompassing networking, flexibility in the use of funds, feasible requirements, a simple but effective application process, and the connection of the fellowship with ASTMH. However, there is room for improvement. A major goal of the fellowship is to forge connections between past and future leaders in TMGH, and the respondents’ call for more interfellow and TMGH networking as well as more extensive follow-up and career mentorship demonstrates that this aspect of the Kean fellowship could be improved on. In response to these comments, ASTMH has sponsored a reception at the Society’s annual meeting for fellows and Kean alumni and offered free registration to the ASTMH meeting for fellows. In recent years, ASTMH has also organized a conference call that includes newly awarded fellows and recent alumni, giving the new cohort a chance to virtually meet each other as well as gain insight and advice from those who have recently completed their international experience. These efforts have seen an increase in attendance by Kean fellows at the ASTMH meeting, an increase in ASTMH membership by Kean fellows, and some increase in networking among fellows. Future efforts should continue to work on expanding ASTMH involvement and networking by fellows, though all such efforts inevitably run into challenges with resource and personnel limitations.

The survey had a number of limitations, including the response rate of 50%. Though this is a respectable response rate for a survey of individuals often still in medical school, residency, or fellowship, it is possible that the 50% who did not respond to the survey were also less likely to remain involved in TMGH. In addition, students who apply for the fellowship already have shown a strong interest in TMGH, and might work and remain in the field whether they received the fellowship. In this regard, the very high rate of responders stating that the Kean fellowship helped them to prepare to pursue a career in TMGH and to network and identify opportunities in the field speaks to the value of the fellowship. Finally, most fellows were still in training, so the long-term effects of the fellowship were not fully assessed. Future surveys, now that the fellowship is approaching its 20th year, should request information specifically from alumni of > 10 years, who should almost all have completed training, to determine the effect of the fellowship on long-term work in TMGH. Of note, one Kean alumnus recently was awarded the Society’s Bailey Ashford Medal for midcareer contributions to research in tropical medicine, demonstrating that the fellowship already has some highly accomplished alumni.

In summary, over the first 15 years of its existence, the Kean fellowship has delivered on its goal of inspiring interest in TMGH in North American medical students and encouraging long-term careers and work in TMGH research and clinical care by these students. As one of the oldest fellowships supporting short-term work by North American medical students in TMGH, and still one of very few such fellowships, it deserves continued support by the Society and its members, as it is helping to build the future pipeline of clinicians and researchers in TMGH.

## Supplementary Material

Supplemental Information.
