# Supplementary material for: The Benjamin H. Kean Travel Fellowship in Tropical Medicine: Assessment of Impact at 15 Years
Source: Am J Trop Med Hyg. 2017 Jun 26;97(3):958–63. doi: 10.4269/ajtmh.17-0120 (PMC5590605; doi:10.4269/ajtmh.17-0120)
Supplement: Supplementary file 1 [file tpmd170120.SD1.pdf]

SUPPLEMENTARY INFORMATION

BENJAMIN H. KEAN TRAVEL FELLOWSHIP ALUMNI SURVEY

1. What is your current job title and institution? (open field response)
2. Are you currently working in tropical medicine/global health, as defined by working in any of the areas below?

Volunteer	Research
Education	Clinical
Public health	Nonprofit/nongovernmental organization (NGO)
Government	Philanthropy
International organization (e.g. World Health Organization [WHO], United Nations High Commissioner for Refugees [UNHCR])	Refugee health care
Mission	Military
Indian Health Service	Immigrant health

3. If you answered yes to question 2, please check all that apply:

Volunteer	Research
Education	Clinical
Public health	Nonprofit/NGO
Government	Philanthropy
International organization (e.g. WHO, UNHCR)	Refugee health care
Mission	Military
Indian Health Service	Immigrant health

4. Are you currently working internationally?

Yes	No

5. If yes, the location is: (open field response)
6. In the time between completing your Kean Fellowship and your current position, did you work in tropical medicine/global health?

Yes	No

7. If you answered yes to the previous question, please check all areas that apply to your work during that time:

Volunteer	Research
Education	Clinical
Public health	Nonprofit/NGO
Government	Philanthropy
International organization (e.g. WHO, UNHCR)	Refugee health care
Mission	Military
Indian Health Service	Immigrant health

8. Was any of your tropical medicine/global health work during this time done internationally?

Yes	No

9. If yes, please indicate location: (open field response)
10. If you answered yes to question 6, what was the total duration of your tropical medicine/global health international work?

1 week or less	2–4 weeks	1–6 months	> 1 year	> 2 years

11. The ASTMH Benjamin Kean Fellowship helped prepare me to pursue a career in tropical medicine/global health.

Strongly agree	Agree	Neutral disagree	Strongly disagree

12. Because of my participation in the ASTMH Benjamin Kean Fellowship, I was better positioned to identify career opportunities in tropical medicine/global health.

Strongly agree	Agree	Neutral disagree	Strongly disagree

13. My participation in the ASTMH Benjamin Kean Fellowship increased or helped me create a network in tropical medicine/global health.

Strongly agree	Agree	Neutral disagree	Strongly disagree

14. My participation in the ASTMH Benjamin Kean Fellowship further inspired me to pursue a career in tropical medicine/global health.

Strongly agree	Agree	Neutral disagree	Strongly disagree

15. My participation in the ASTMH Benjamin Kean Fellowship helped me to identify funding opportunities in tropical medicine/global health.

Strongly agree	Agree	Neutral disagree	Strongly disagree

16. My participation in the ASTMH Benjamin Kean Fellowship helped me to secure funding opportunities in tropical medicine/global health.

Strongly agree	Agree	Neutral disagree	Strongly disagree

17. In your opinion, what were the strengths of the ASTMH Benjamin Kean Fellowship? (open field response)
18. In your opinion, what aspects or components of the ASTMH Benjamin Kean Fellowship should definitely not be changed? (open field response)
19. What suggestions do you have to improve the program for future participants? (open field response)
